# Adhesion of resin cement to 3D printed dental polymers: effect of layer thickness and air abrasion

**DOI:** 10.1007/s00784-026-06936-2

**Published:** 2026-05-30

**Authors:** Paula Zwicky, Michele Piccini, Abdülhakim Alpaslan Sener, Luiza Freitas Brum Souza, Tan Fırat Eyüboğlu, Mutlu Özcan

**Affiliations:** 1 Center for Dental Medicine, Clinic of Masticatory Disorders and Dental Biomaterials, University of Zurich, Plattenstrasse 11, CH-8032 Zurich, Switzerland; 2 Faculty of Dentistry, Department of Endodontics, Istanbul Medipol University, Istanbul, Türkiye

**Keywords:** 3D-printing, Additive manufacturing, Adhesion, Dental materials, Fixed dental prosthesis, Resin Composite, Thermocycling, Prosthetic Dentistry

## Abstract

**Objectives:**

To assess the influence of layer thickness, silica-coating, and aging on surface roughness and resin cement adhesion to 3D-printed dental polymers.

**Materials and methods:**

Four photo-polymerized resins (Crowntec (S), 3Delta Etemp (D), TempPrint (GC), and NextDent C&B MFH (ND)) were printed under four layer conditions (polished, 20, 50, and 100 µm; *N* = 1280). Specimens were treated with or without silica-coating air abrasion (CoJet), bonded with resin cement (Panavia V5), and tested dry or after thermocycling (5,000 cycles). Surface roughness (Ra, Rz) and shear bond strength (SBS) were analyzed using three-way ANOVA and Tukey’s test (α = 0.05).

**Results:**

Material, layer thickness, and surface conditioning significantly affected surface roughness (*p* < 0.001), with material- and layer-dependent responses to silica coating. Roughness generally increased with layer thickness. For SBS, material and layer thickness were significant under both conditions (*p* < 0.05), while silica coating mainly benefited polished surfaces. ND and GC showed the highest SBS at 20 µm, S exhibited stable performance, and D showed the lowest bond strength. Thermocycling did not consistently reduce adhesion.

**Conclusions:**

Printing layer thickness and material composition are key determinants of surface roughness and bonding. Silica-coating was most effective on polished surfaces, and optimal adhesion was achieved with ND and GC printed at 20 µm.

**Clinical relevance:**

Tailoring printing resolution and surface conditioning improves adhesion to 3D-printed resin restorations.

## Introduction

Dental resin composites are vital in prosthodontics for their ease of handling, intraoral reparability, and aesthetics [1, 2]. Originally used for direct restorations, their applications have expanded to indirect uses with advances in computer-aided design/manufacturing (CAD-CAM) and additive manufacturing (AM) technologies [3, 4]. AM, or 3D printing, enables precise, efficient fabrication with less material waste [5]. Stereolithography (SLA) is prominent in dentistry for creating detailed restorations from ceramic-filled photopolymerizing resin through layer-by-layer photopolymerization. These methods produce crowns, frameworks, and both provisional and final restorations, meeting the demand for minimally invasive and esthetic prostheses [5].

Factors influencing 3D printed resin performance include printing parameters, especially layer thickness, which significantly affect surface topography, energy, and adhesion potential [6–8]. Layer thickness determines vertical resolution and impacts surface irregularities: smaller layers yield smoother, more uniform surfaces that enhance light diffusion, while thicker layers create visible stair-steps, increasing roughness and potentially reducing adhesion [9, 10]. Surface roughness is crucial for adhesion, as it influences wetting, micromechanical interlocking, and interactions with bonding agents’ functional groups [11, 12].

Surface features influence how adhesives bond, so surface conditioning is key for durability [13, 14]. Air abrasion with silica-coating is effective for resin and hybrid materials [15, 16], creating roughness and a silica layer for improved adhesion [17, 18]. In printed resins, this dual mechanism is especially important due to the inert, low-energy surface of the photopolymerized matrix [16, 19]. The air-abrasion parameters used in this study (30 µm particles, 2.5 bar pressure, 10 mm working distance, and 5 s application time) correspond to commonly recommended silica-coating protocols and are compatible with chairside surface conditioning procedures, supporting the clinical relevance of the experimental conditions [15, 17].

Adhesion studies using shear bond strength (SBS) tests are widely used to evaluate interfacial performance between resin cements and restorative materials under controlled conditions [20, 21]. These tests simulate clinical stress distributions and assess how surface treatments and printing parameters affect adhesive behavior [22]. Moreover, thermocycling and water storage are essential for reproducing intraoral aging, as repeated temperature changes induce interfacial stresses and hydrolytic degradation, potentially compromising bond durability [22–24]. Nevertheless, thermocycling may also promote additional polymer cross-linking and post-polymerization, thereby enhancing interfacial stability in some materials [19]. Despite the growing use of 3D-printed resins in prosthodontics, limited information is available on how layer thickness and surface pretreatments influence adhesion and durability after aging [14, 16].

In this context, this study aimed to evaluate the shear bond strength and surface roughness of 3D-printed resin composites manufactured with different layer thicknesses, including a polished control group, with half of the specimens subjected to air abrasion prior to cementation using the same resin cement. Additionally, surface roughness and topography were assessed. The null hypotheses tested were that 1) different 3D-printing resin materials; 2) different printed layer thicknesses; and 3) air abrasion with silica-coating would not affect the surface topography and adhesion of 3D-printed resins.

## Materials and methods

### Materials and study design

**Fig. 1:**
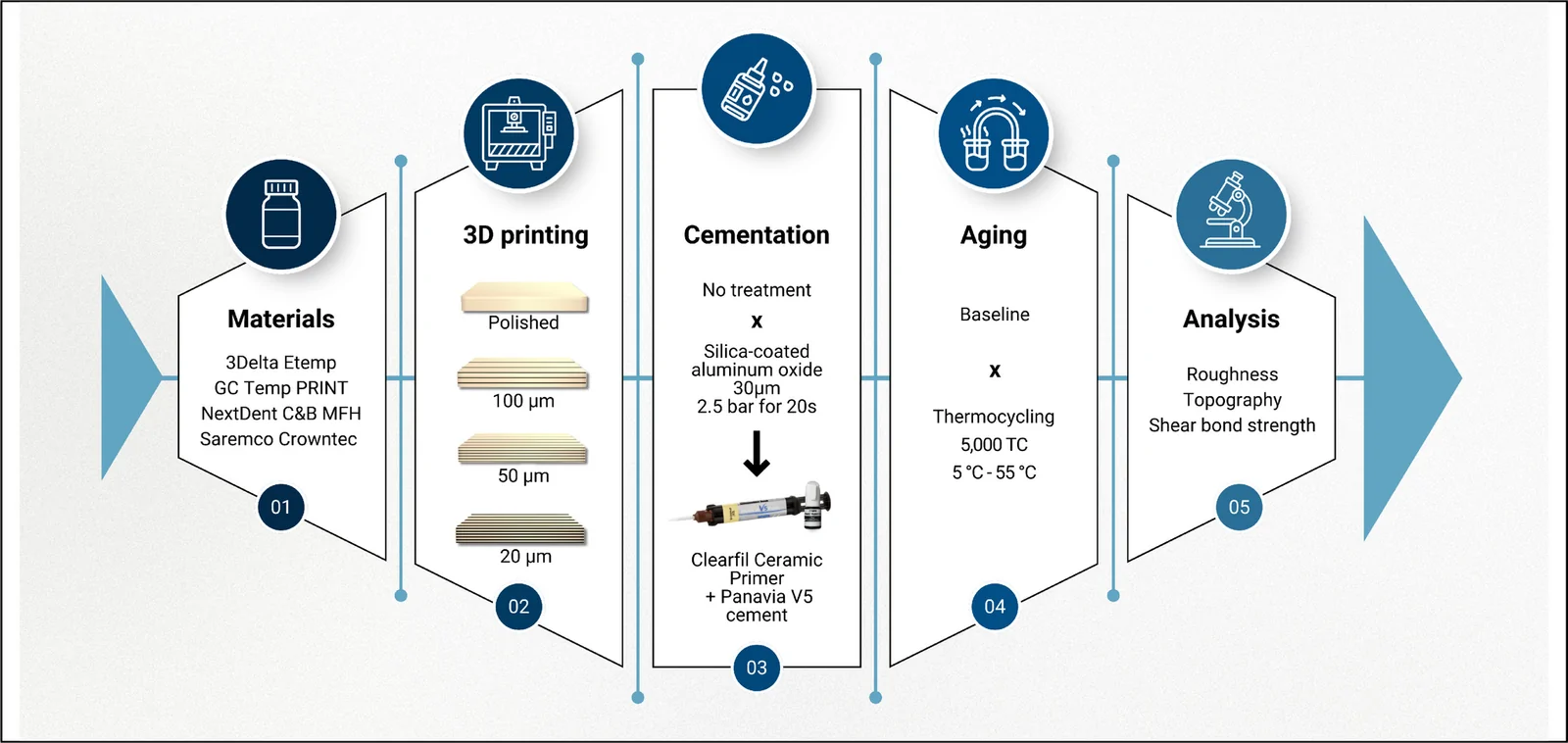
Flowchart illustrating the study design

This study employed a 4 × 4 × 2 × 2 factorial design to investigate the effects of material type (Crowntec, SAREMCO Dental; 3Delta Etemp, DeltaMed; TempPrint, GC; and NextDent C&B MFH, Vertex-Dental), layer thickness (polished, 20 µm, 50 µm, and 100 µm), surface conditioning (no treatment or silica-coating; CoJet, 30 µm, 5 s, 2.5 bar), and aging condition (dry or thermocycling) (Fig. 1). A total of 1280 specimens were distributed across the experimental groups, resulting in 20 specimens per condition.

Table 1 provides a detailed summary of the materials used, including their brands, manufacturers, chemical compositions, and batch numbers. Among the evaluated printable resins, 3Delta Etemp and GC Temp PRINT are primarily indicated for provisional crown and bridge restorations, whereas NextDent C&B MFH and Saremco Crowntec are indicated for long-term or definitive prosthetic restorations. The primary response variable was shear bond strength (SBS). Additional evaluations included surface roughness and topography.

**Table 1:** Description of brands, manufacturers, chemical composition and batch numbers of materials used

Material	Manufacturer	Chemical Composition	Batch numbers
3Delta Etemp (D)	Deltamed, Friedberg, Germany	Methacrylates, urethane dimethacrylate (UDMA), trimethylolpropane triacrylate (TMPTA), Silicon dioxide (SiO_2_) 50 wt%, dental glass 30 vol%, Diphenyl(2,4,6-trimethylbenzoyl)phosphine oxide (TPO)	2311010
GC Temp PRINT™ (GC)	GC Europe AG, Leuven, Belgium​	Urethane dimethacrylate (UDMA, 50–75 wt%), 2,2'-ethylenedioxyethyl dimethacrylate (10–25 wt%), esterification products of 4,4'-isopropylidenediphenol with ethoxylated and 2-methylprop-2-enoic acid (2.5–5 wt%),Quartz (SiO_2_, 10–25 wt%), Diphenyl(2,4,6-trimethylbenzoyl)phosphine oxide (1– < 2.5 wt%), UV absorber (2-(2H-benzotriazol-2-yl)-p-cresol, 0.1– < 0.2 wt%)	2501171
NextDentTC&B MFH (ND)	NextDent, Soesterberg, Netherlands	Methacrylic oligomers (> 60 wt%), glycol methacrylate (15–25 wt%),Phosphine oxide (< 2.5 wt%)	WU4S4N04
Saremco Crowntec (S)	Saremco Dantal AG, Rebstein, Switzerland	Bisphenol A ethoxylated dimethacrylate (Bis-EMA, 50– < 75 wt%), Bisphenol A polyethylene glycol diether dimethacrylate (50– < 75 wt%), Methyl benzoylformate (1– < 5 wt%), diphenyl(2,4,6-trimethylbenzoyl)phosphine oxide (0.1– < 5 wt%)	F629
CoJet	3 M ESPE, St. Paul, MN, USA	Aluminum oxide (> 95 wt%), synthetic amorphous silica (< 5 wt%), titanium dioxide (< 0.6 wt%)	10579307
K-ETCHANT Syringe	Kuraray Noritake Dental Inc., Okayama, Japan	Phosphoric acid (35 wt%), water, colloidal silica, pigments	5R0167
Clearfil Ceramic Primer	Kuraray Medical Inc., Okayama, Japan	Ethanol, 3-trimethoxysilylpropyl methacrylate (silane), 10-methacryloyloxydecyl dihydrogen phosphate (MDP)	6C0096
Panavia V5	Kuraray Medical Inc., Okayama, Japan	Bis-GMA (5–15 wt%), triethylene glycol dimethacrylate (TEGDMA, < 5 wt%), hydrophobic aromatic dimethacrylate, hydrophobic aliphatic dimethacrylate, silanated barium glass filler, silanated fluoroaluminosilicate glass filler, silanated alumina filler, amorphous silica, camphorquinone (CQ), accelerators, initiators, pigments	630080 350274

^*^UDMA urethane dimethacrylate, *HEMA* 2-hydroxyethylmethacrylate, *Bis-GMA* bisphenol-A-gycidyl dimethacrylate, *TEGDMA* triethylenglycol dimethacrylate

### Specimen preparation

Computer-Aided Design (CAD) (Exocad DentalCAD software) was used to design 3D-printed resin specimens in a square shape (15 mm × 15 mm × 2 mm), providing a standardized flat bonding surface suitable for shear bond strength testing in accordance with commonly adopted ISO testing principles for dental adhesive interfaces (ISO 29022) [25]. Three layer thicknesses were defined: 20 µm, 50 µm, and 100 µm, representing printing resolutions commonly used in dental additive manufacturing and corresponding to the practical lower and upper limits applied in routine clinical laboratory workflows. Specimens fabricated at 100 µm were subsequently polished. The specimens were additively manufactured using 3D printing technology (Phrozen Sonic Mini Resin 8 K 3D Printer) with four different photopolymerized resins, corresponding to the comparative groups (D: 3Delta Etemp, GC: GC Tempprint, ND: NextDentTM C&B MFH, and S: Saremco Crowntec).

After printing, specimens were ultrasonically cleaned for 5 min (Anycubic Wash & Cure 2.0, Shenzhen, China) with 99% ethanol to remove excess resin monomers. They were then post-polymerized for 15 min in a photopolymerization unit (SprintRay ProCure 2, Los Angeles, USA), using a manufacturer-recommended photo-polymerization program designed for dental photopolymer resins, which ensures adequate post-curing and promotes the formation of a stable cross-linked polymer network.

For the polished group, the specimens were wet-ground with silicon carbide paper (#400 and #1200 grit) under constant water cooling (Labpool 21, Struers GmbH, Copenhagen, Denmark) until a smooth, polished surface was achieved. Subsequently, the specimens were cleaned in an ultrasonic bath (Telesonic TPC-25, Bronschhofen, Switzerland) in distilled water for 10 min, then air-dried.

### Cementation procedure and artificial aging

Before bonding, half of the specimens in each group were subjected to silica coating using 30 µm silica-coated alumina particles (Co-Jet Sand, 3 M ESPE, St. Paul, USA) applied for 5 s at a pressure of 2.5 bar and a working distance of 10 mm. After air-abrasion, residual particles were removed with an air spray. Specimens were then cleaned in an ultrasonic bath with distilled water for 2 min and dried with an air spray. An etchant (K-ETCHANT Syringe, Kuraray Noritake Dental Inc., Okayama, Japan) was applied for 5 s, followed by thorough rinsing with water and air-drying. Subsequently, a silane coupling agent (Clearfil Ceramic Primer Plus, Kuraray Noritake Dental Inc., Okayama, Japan) was applied according to the manufacturer’s instructions.

Transparent polyethylene molds (height: 4 mm; internal diameter: 3 mm) were placed on the conditioned surfaces with a custom-made holder. Resin cement (Panavia V5, Kuraray Noritake Dental Inc., Okayama, Japan) was applied inside the molds in two increments and photo-polymerized for 20 s each (Bluephase Style, Ivoclar AG, Schaan, Liechtenstein) at an intensity of 1000 mW/cm^2^. This adhesive resin cement was selected because it is widely used for the cementation of indirect restorations such as crowns and bridges. All bonding procedures were performed by a single calibrated operator to ensure consistency.

Each conditioned group was randomly divided into two subgroups based on the aging protocol: Dry and Thermocycling (TC). All specimens were initially stored dry in an incubator at 37 °C for 24 h. The TC group was then subjected to artificial aging consisting of 5000 thermal cycles between 5 °C and 55 °C, with a dwell time of 20 s in each bath and a transfer time of 5 s. The thermocycling process concluded in the cold bath to standardize conditions prior to testing.

### Roughness and topography analysis

After the air-abrasion procedure and before cementation, micrometric roughness analysis was performed for each specimen using a 3D digital microscope (VHX 7000, Keyence Co., Osaka, Japan). Profile lines were extracted from the 3D images to determine the difference between the smallest and largest undulations, which were then reported as roughness. Six measurements, three along each axis (*x* and *y*), were taken with a cutoff (λc) of 0.25 mm and a sampling length of 1.25 mm. The arithmetic mean was calculated for each specimen following ISO 21920–2 (2021), using the Ra (average surface roughness) and Rz (maximum peak-to-valley height) parameters. In addition, digital images were captured at 100 × magnification for each material and layer thickness, before and after the surface treatment.

### Bond strength test and failure analysis

**Fig. 2:**
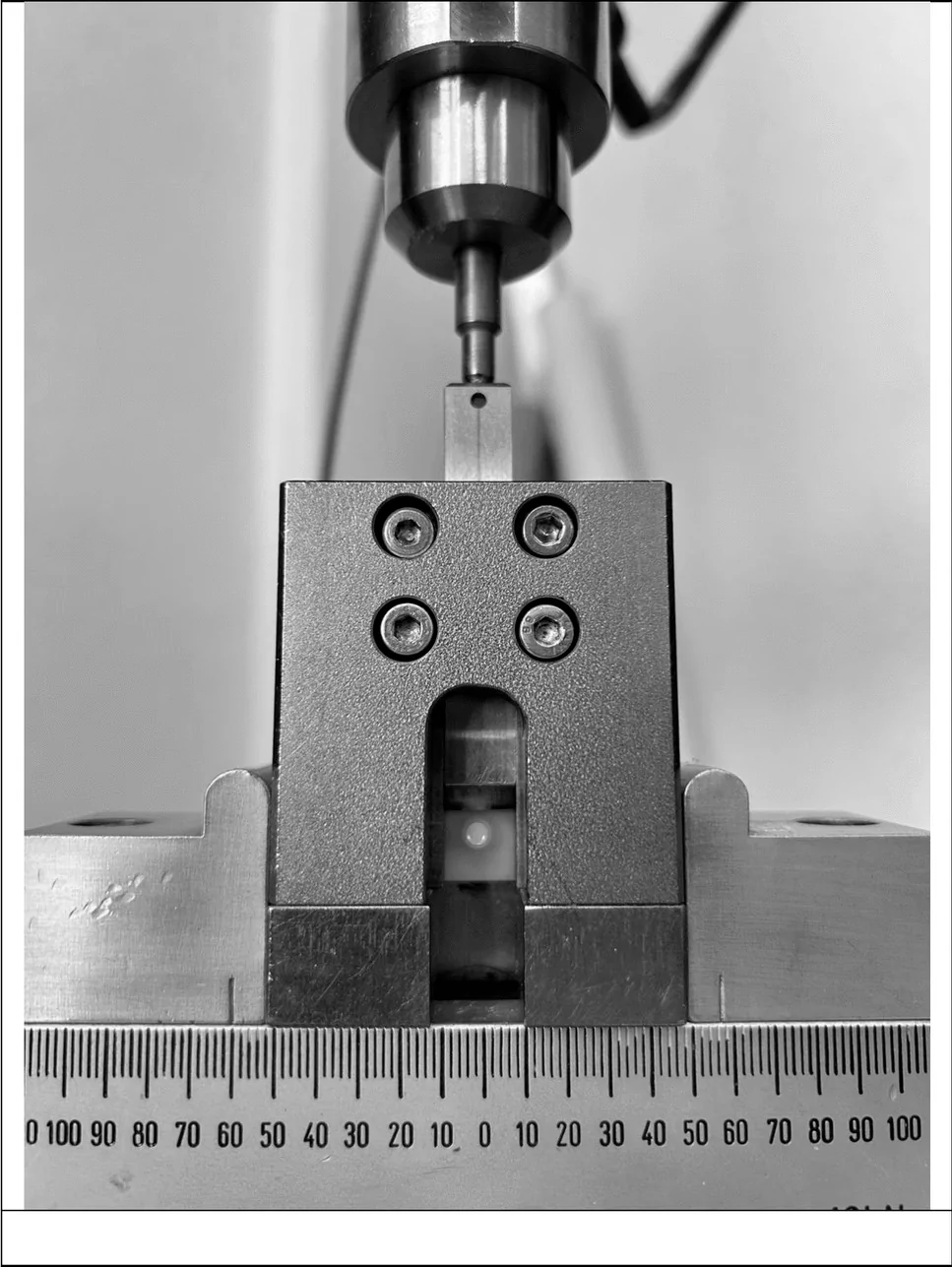
Shear bond strength test setup

Specimens were fixed in a jig of a universal testing machine (Zwick/Roell Z2.5 MA 18–1–3/7, Ulm, Germany), and shear forces were applied to the adhesive interface with a shearing blade until debonding occurred (Fig. 2). The load was applied as close as possible to the bonded interface at a crosshead speed of 1 mm/min. Shear bond strength (SBS) values (MPa) were calculated using the formula S = L/A, where S is the bond strength (MPa), L is the load at failure (N), and A is the bonded area (mm^2^).

After testing, debonded surfaces were examined under a digital microscope (VHX-7000, Keyence Co., Osaka, Japan) at 50 × magnification to determine the failure mode. Failure types were classified as follows: Type 0: Adhesive failure: complete detachment at the substrate-cement interface, with no cement remnant visible on the substrate; Type 1a: Predominantly adhesive failure (< 1/3 cement remnant): a limited remnant covering less than one-third of the bonded area. This remnant may consist of cement and/or cohesive fracture of the resin substrate; Type 1b: a remnant covering more than two-thirds of the bonded area. This may correspond to cement remnants and/or cohesive fracture within the resin substrate; Type 2: Cohesive failure: fracture occurs within the cement and/or within the resin specimen (substrate), with the interface remaining intact.

### Statistical analysis

Statistical analyses were performed using SPSS (version 21.0; IBM Corp., Armonk, NY, USA). Roughness and shear bond strength data exhibited homoscedasticity (Levene’s test, *p* > 0.05) and normal distribution (Shapiro–Wilk test, *p* > 0.05). A three-way analysis of variance (ANOVA) was performed to evaluate the effects of material, layer thickness, and surface conditioning, as well as their interactions, on surface roughness and SBS, separately for Dry and Thermocycling (TC) conditions. Subsequently, a two-way ANOVA with Tukey’s post hoc test (α = 0.05) was used to compare the effects of layer thickness and surface conditioning within each material. For shear bond strength, the aging effect was analyzed separately.

Weibull statistical analysis was performed using SuperSMITH Weibull software (version 4.0 k-32; Wes Fulton, Torrance, CA, USA) to assess the reliability and predictability of bond strength values for each combination of material, layer thickness, and surface conditioning. A two-parameter Weibull distribution was fitted to the failure-load data (converted to MPa), yielding the characteristic strength (η), the stress at which 63.2% of specimens are predicted to fail, and the Weibull modulus (β), which describes the distribution of failures and indicates reliability. Higher β values reflect less variability and greater reliability of bonding performance.

Topographic characteristics and failure modes were qualitatively and descriptively analyzed.

## Results

The three-way ANOVA showed that material, layer thickness, and surface conditioning each had significant main effects on both Ra and Rz (*p* < 0.001). In addition, significant two-way interactions (Material × Layer, Material × Treatment, and Layer × Treatment) and a significant three-way interaction (Material × Layer × Treatment) were observed for both roughness parameters (*p* < 0.001), indicating that although silica coating strongly altered surface roughness overall, the magnitude and pattern of this effect depended on the specific material and printing layer thickness (Table 2).

**Table 2:** Mean and standard deviations for surface roughness parameters – Ra and Rz (in µm)

Material	Layer thickness	Surface treatment	Ra (µm) Mean ± SD	Rz (µm) Mean ± SD
D	Polished	No treatment	0.66 ± 0.12 ^D^	3.83 ± 0.97 ^B^
		Silica coating	20.98 ± 0.36 ^A^	121.43 ± 5.61 ^A^
	20 µm	No treatment	8.60 ± 0.64 ^C^	35.06 ± 2.26 ^B^
		Silica coating	23.78 ± 0.99 ^A^	136.20 ± 2.39 ^A^
	50 µm	No treatment	11.00 ± 2.64 ^BC^	96.38 ± 2.87 ^A^
		Silica coating	23.01 ± 1.28 ^A^	131.17 ± 9.99 ^A^
	100 µm	No treatment	13.53 ± 2.12 ^B^	136.47 ± 22.45 ^A^
		Silica coating	19.39 ± 2.62 ^A^	114.01 ± 19.61 ^A^
ND	Polished	No treatment	5.50 ± 1.18 ^B^	48.40 ± 6.70 ^B^
		Silica coating	22.09 ± 2.61 ^A^	134.08 ± 10.34 ^A^
	20 µm	No treatment	23.27 ± 1.16 ^A^	132.31 ± 9.80 ^A^
		Silica coating	20.02 ± 0.86 ^A^	120.21 ± 2.74 ^A^
	50 µm	No treatment	20.57 ± 1.67 ^A^	117.04 ± 11.95 ^A^
		Silica coating	22.10 ± 0.60 ^A^	130.35 ± 5.13 ^A^
	100 µm	No treatment	6.69 ± 0.61 ^B^	103.12 ± 27.34 ^A^
		Silica coating	22.92 ± 1.31 ^A^	131.74 ± 2.78 ^A^
GC	Polished	No treatment	7.14 ± 0.07 ^D^	33.33 ± 6.84 ^C^
		Silica coating	20.86 ± 1.56 ^C^	123.13 ± 7.64 ^B^
	20 µm	No treatment	22.24 ± 1.30 ^C^	130.35 ± 3.27 ^B^
		Silica coating	36.89 ± 2.77 ^A^	168.89 ± 0.98 ^A^
	50 µm	No treatment	22.60 ± 0.87 ^C^	145.87 ± 21.00 ^AB^
		Silica coating	22.92 ± 0.72 ^C^	130.36 ± 9.44 ^B^
	100 µm	No treatment	24.51 ± 1.30 ^C^	137.52 ± 10.63 ^AB^
		Silica coating	30.82 ± 1.66 ^B^	152.26 ± 19.21 ^AB^
S	Polished	No treatment	1.79 ± 0.25 ^C^	10.98 ± 5.56 ^B^
		Silica coating	17.54 ± 0.06 ^B^	113.75 ± 5.72 ^A^
	20 µm	No treatment	19.79 ± 1.11 ^AB^	114.74 ± 8.97 ^A^
		Silica coating	20.55 ± 1.06 ^A^	124.48 ± 4.31 ^A^
	50 µm	No treatment	20.97 ± 0.81 ^A^	126.99 ± 4.55 ^A^
		Silica coating	17.57 ± 1.00 ^B^	105.18 ± 12.89 ^A^
	100 µm	No treatment	21.05 ± 0.77 ^A^	124.33 ± 10.38 ^A^
		Silica coating	20.16 ± 0.71 ^A^	115.19 ± 8.56 ^A^

^*^Different uppercase superscript letters (A, B, C, D) indicate statistically significant differences among the evaluated conditions according to two-way ANOVA (layer thickness and surface treatment within each material) followed by Tukey’s HSD post hoc test (α = 0.05)

According to the two-way ANOVA and post-hoc comparisons, material D showed a progressive increase in Ra and Rz with increasing layer thickness under untreated conditions, with polished surfaces exhibiting the lowest roughness, followed by 20 µm, 50 µm, and 100 µm layers. After silica coating, Ra and Rz increased markedly across all layer thicknesses, resulting in similar roughness values under printed and polished conditions.

For material ND, the untreated polished and 100 µm groups showed the lowest roughness, whereas the untreated 20 µm and 50 µm groups already exhibited high Ra and Rz values. Silica coating significantly increased roughness in the polished and 100 µm groups but produced no substantial additional increase in the already rough 20 µm and 50 µm groups.

For GC material, the untreated polished surface exhibited the lowest Ra and Rz, while the untreated printed groups showed higher roughness values. Following silica coating, roughness increased in most groups, except for the 50 µm condition, which showed similar Ra values before and after treatment. The highest roughness values after silica coating were observed at 20 µm, followed by 100 µm, 50 µm, and polished surfaces.

For material S, the untreated polished group again showed the lowest Ra and Rz, whereas the untreated printed groups exhibited comparably higher roughness values. After silica coating, Ra increased primarily for the polished condition, decreased for the 50 µm group, and remained largely unchanged for the 20 µm and 100 µm groups. For Rz, the silica coating yielded similarly high values across all layer thicknesses, with the untreated polished surface the only condition with distinctly lower Rz.

For SBS (Table 3), in the dry condition, material (*p* < 0.0001) and layer thickness (*p* = 0.0023) showed significant main effects, whereas surface conditioning did not (*p* = 0.1554). The descriptive results (mean ± SD) for shear bond strength measurements across all materials, layer thicknesses, surface treatments, and aging conditions are presented in Table 4. However, the three-way interaction among material, layer thickness, and surface conditioning was significant (*p* < 0.0001), indicating condition-specific effects of silica coating.

**Table 3:** Three-way ANOVA results for shear bond strength (SBS)

Source	DF	F	P
*Dry*			
Material	3	125.23	0.0000*
Layer	3	4.96	0.0023*
Treatment	1	2.03	0.1554
Material*Layer	9	10.96	0.0000*
Material*Treatment	3	1.38	0.2503
Layer*Treatment	3	2.53	0.0577
Material*Layer*Treatment	9	4.65	0.0000*
*Thermocycling*			
Material	3	180.72	0.0000*
Layer	3	3.91	0.0092*
Treatment	1	1.86	0.1731
Material*Layer	9	15.75	0.0000*
Material*Treatment	3	5.98	0.0006*
Layer*Treatment	3	3.08	0.0278*
Material*Layer*Treatment	9	2.33	0.0150*

**Table 4:** Results (mean and standard deviations) for Shear Bond Strength (SBS) test in MPa

Material	Layer thickness	Surface treatment	Dry	TC	% decrease between Dry and TC means
			SBS (MPa)* Mean ± SD	SBS (MPa)* Mean ± SD	
D	Polished	No treatment	2.32 ± 0.80 ^B^	3.04 ± 1.87 ^A^	−31.03
		Silica coating	8.08 ± 2.06 ^A^	3.62 ± 1.79 ^A^	55.20
	20 µm	No treatment	3.14 ± 1.80 ^B^	2.70 ± 0.73 ^A^	14.01
		Silica coating	2.31 ± 0.92 ^B^	2.79 ± 1.40 ^A^	−20.78
	50 µm	No treatment	2.88 ± 0.75 ^B^	3.27 ± 0.97 ^A^	−13.54
		Silica coating	3.86 ± 2.83 ^B^	3.14 ± 2.31 ^A^	18.65
	100 µm	No treatment	3.09 ± 1.64 ^B^	2.01 ± 0.41 ^A^	34.95
		Silica coating	3.95 ± 1.51 ^B^	2.53 ± 1.79 ^A^	35.95
ND	Polished	No treatment	7.35 ± 2.83 ^CD^	5.44 ± 2.58 ^B^	25.99
		Silica coating	6.42 ± 2.04 ^D^	3.12 ± 1.51 ^B^	51.40
	20 µm	No treatment	15.20 ± 6.52 ^A^	12.36 ± 2.92 ^A^	18.68
		Silica coating	16.87 ± 2.78 ^A^	11.39 ± 2.46 ^A^	32.48
	50 µm	No treatment	13.96 ± 2.39 ^AB^	10.34 ± 3.15 ^A^	25.93
		Silica coating	12.18 ± 3.99 ^ABC^	9.34 ± 2.68 ^A^	23.32
	100 µm	No treatment	9.81 ± 3.04 ^BCD^	11.68 ± 2.29 ^A^	−19.06
		Silica coating	10.00 ± 1.68 ^BCD^	9.76 ± 2.85 ^A^	2.40
GC	Polished	No treatment	9.49 ± 3.25 ^BCD^	6.46 ± 2.29 ^D^	31.93
		Silica coating	12.31 ± 3.27 ^AB^	13.45 ± 4.81 ^A^	−9.26
	20 µm	No treatment	15.14 ± 2.97 ^A^	9.78 ± 1.66 ^ABCD^	35.40
		Silica coating	9.23 ± 2.33 ^BCD^	9.10 ± 2.59 ^BCD^	1.41
	50 µm	No treatment	6.10 ± 2.38 ^D^	6.60 ± 2.69 ^CD^	−8.20
		Silica coating	10.46 ± 1.89 ^BC^	7.19 ± 1.99 ^CD^	31.26
	100 µm	No treatment	9.45 ± 2.86 ^BCD^	10.60 ± 2.63 ^ABC^	−12.17
		Silica coating	8.00 ± 3.98 ^CD^	11.47 ± 3.28 ^AB^	−43.38
S	Polished	No treatment	13.91 ± 4.39 ^A^	14.07 ± 4.69 ^AB^	−1.15
		Silica coating	12.55 ± 4.75 ^A^	16.58 ± 4.78 ^A^	−32.11
	20 µm	No treatment	11.02 ± 3.64 ^A^	10.48 ± 2.81 ^B^	4.90
		Silica coating	13.59 ± 6.07 ^A^	11.16 ± 2.85 ^B^	17.88
	50 µm	No treatment	11.84 ± 1.44 ^A^	10.53 ± 2.88 ^B^	11.06
		Silica coating	14.32 ± 6.50 ^A^	12.33 ± 2.46 ^AB^	13.90
	100 µm	No treatment	14.73 ± 3.12 ^A^	13.34 ± 2.07 ^AB^	9.44
		Silica coating	13.74 ± 4.20 ^A^	12.34 ± 3.82 ^AB^	10.19

^*^Different uppercase superscript letters (A, B, C, D) indicate statistically significant differences among the evaluated conditions according to two-way ANOVA (layer thickness and surface treatment within each material) followed by Tukey’s HSD post hoc test (α = 0.05)

After thermocycling, material (*p* < 0.0001) and layer thickness (*p* = 0.0092) again showed significant main effects, while surface conditioning remained non-significant as a main effect (*p* = 0.1731). Nevertheless, significant interaction effects were observed, confirming that the influence of silica coating and layer thickness on bond strength depended on material type and aging condition.

For material D in the dry condition, all groups exhibited statistically similar SBS values, except for the polished silica-coating group, which showed significantly higher bond strength than the others. After thermocycling, no statistically significant differences were detected among any combinations of layer thickness and surface treatment.

For material ND, the 20 µm groups, with and without silica coating, exhibited higher SBS values than the polished and 100 µm groups under dry conditions, while the 50 µm groups showed intermediate values. After thermocycling, all printed groups (20, 50, and 100 µm), regardless of surface treatment, showed similar SBS values, whereas both polished conditions exhibited lower bond strength than the printed groups.

For GC material under dry conditions, the 50 µm untreated group had the lowest SBS, while the 20 µm untreated group had the highest values and was statistically similar to several intermediate conditions. After thermocycling, the polished untreated and 50 µm untreated groups had the lowest SBS, whereas the polished silica-coating and 20 µm untreated groups had the highest bond strengths.

For material S, no statistically significant differences were observed among groups under dry conditions. After thermocycling, the polished silica-coating group exhibited higher SBS values than the 20 µm and 50 µm untreated groups, while all other conditions were statistically similar.

**Fig. 3:**
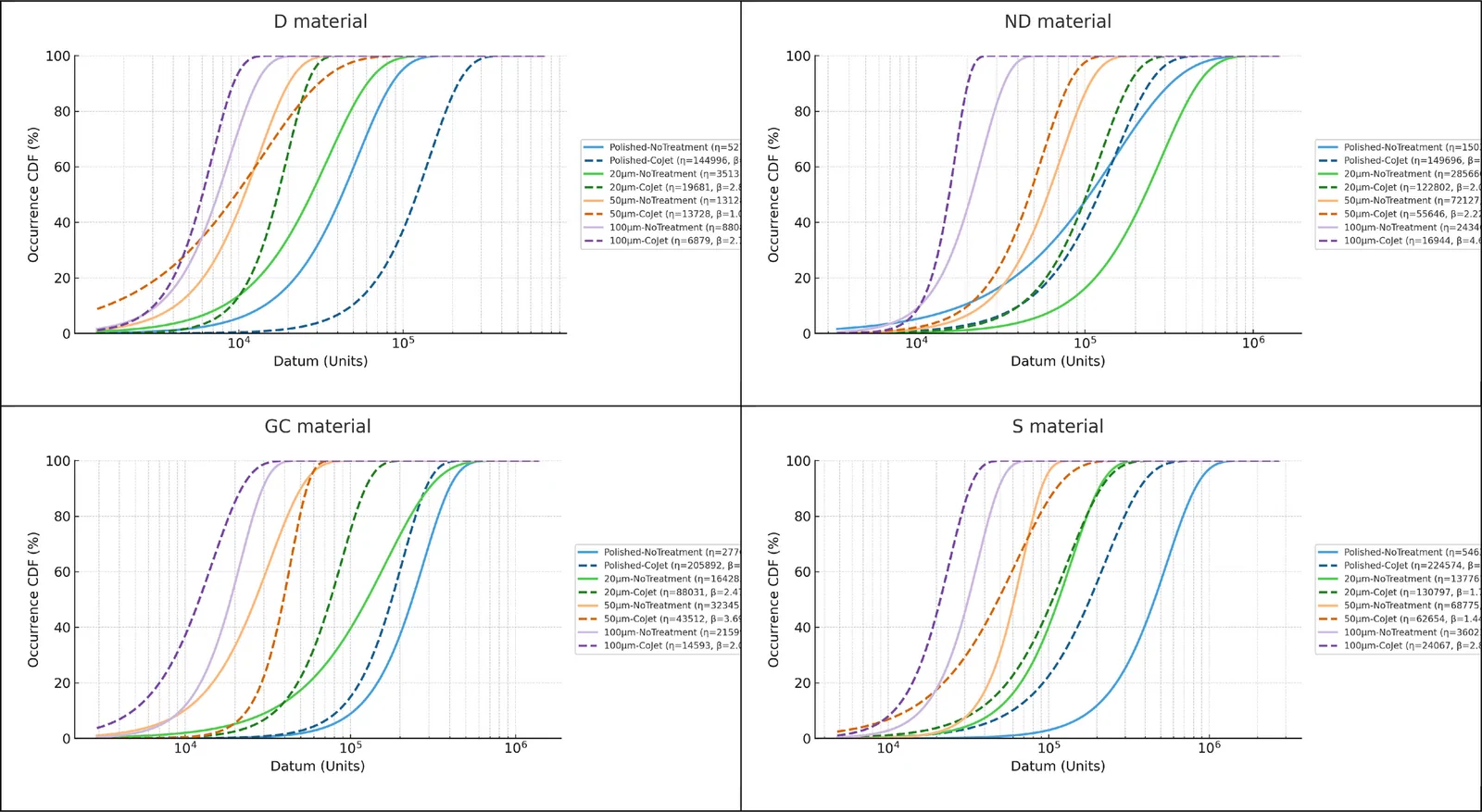
Weibull probability plots showing the cumulative distribution function (CDF) of shear bond strength for all materials under dry conditions. Each plot compares the effect of different layer thickness and surface treatments on the estimated characteristic strength (η) and Weibull modulus (β)

**Fig. 4:**
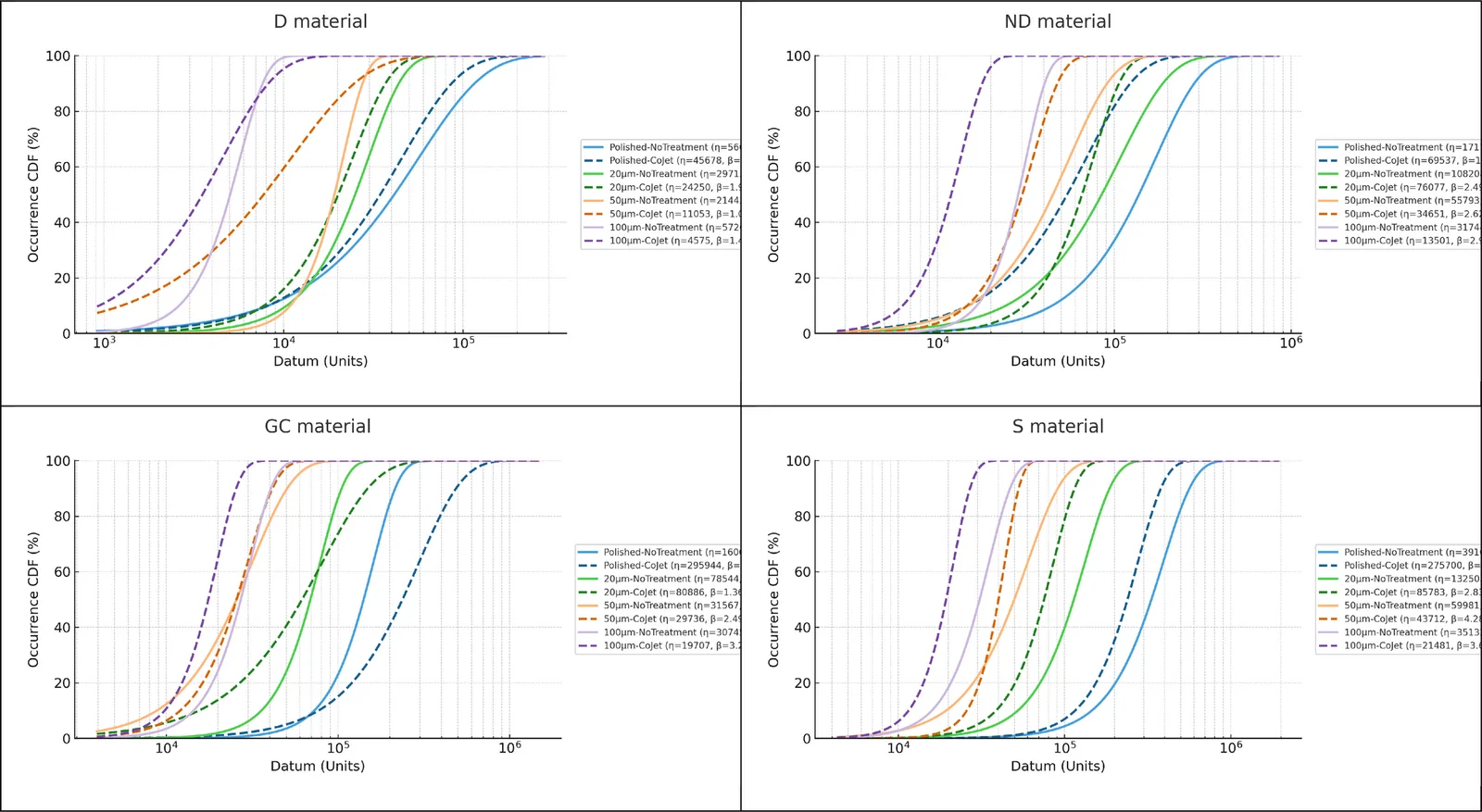
Weibull probability plots showing the cumulative distribution function (CDF) of shear bond strength for all materials under thermocycling conditions. Each plot compares the effect of different layer thickness and surface treatments on the estimated characteristic strength (η) and Weibull modulus (β)

Weibull analysis revealed material- and condition-dependent differences in bond reliability. The Weibull probability plots illustrating the cumulative distribution of shear bond strength under dry and thermocycling conditions are shown in Figs. 3 and 4, respectively. Silica-coating groups frequently exhibited steeper Weibull slopes (higher β values), indicating reduced variability and more predictable failure behavior, whereas untreated groups, particularly polished untreated conditions, showed broader distributions and greater scatter. After thermocycling, several groups exhibited increased Weibull moduli, suggesting reduced variability compared with their dry counterparts.

**Fig. 5:**
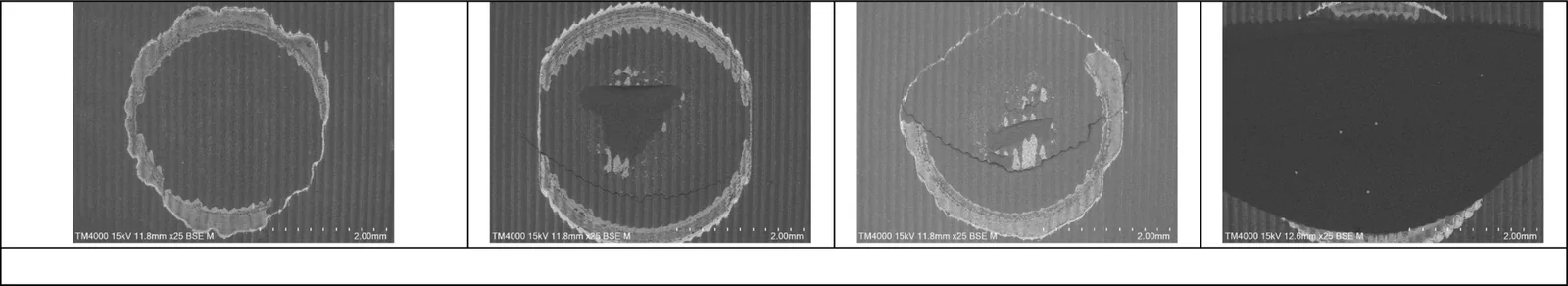
Representative SEM images of failure modes after shear bond strength (SBS) testing. Type 0 (adhesive failure): complete separation at the substrate-cement interface with no residual material. Type 1a (predominantly adhesive): less than one-third of the bonded area covered by remnant, which may include cement and/or cohesive fracture of the resin substrate. Type 1b (predominantly cohesive): more than two-thirds of the bonded area covered by remnant, corresponding to cement and/or cohesive fracture within the resin substrate. Type 2 (cohesive failure): fracture occurring within the cement and/or within the resin substrate, with the interface remaining intact

The distribution of failure modes varied across materials (D, ND, GC, and S), layer thicknesses, and surface treatments. Overall, mixed failures (Types 1a and 1b) and cohesive failures (Type 2) consistently involved fracture within the resin substrate rather than within the cement, indicating that the cement layer remained intact in these cases. Materials exhibiting higher bond strength were associated with a greater prevalence of cohesive failures, whereas materials with lower bond strength showed predominantly adhesive failures (Type 0). Material D showed a higher proportion of adhesive failures, particularly under dry conditions, reflecting lower interfacial strength. In contrast, ND and GC presented a more balanced distribution between adhesive and cohesive failures depending on surface treatment and aging condition. Material S exhibited a clear predominance of cohesive failures across most conditions, indicating superior adhesion, with failure governed primarily by the mechanical limits of the resin substrate rather than the adhesive interface (Table 5, Fig. 5).

**Table 5:** Distribution (%) of failure modes after shear bond strength (SBS) testing for each material, layer thickness, and surface treatment under dry and thermocycling (TC) conditions

Material	Layer thickness	Surface treatment	Dry				TC			
			Type 0 (%)	Type 1a (%)	Type 1b (%)	Type 2 (%)	Type 0 (%)	Type 1a (%)	Type 1b (%)	Type 2 (%)
D	Polished	No treatment	70	20	0	10	60	30	0	10
		Silica coating	80	20	0	0	100	0	0	0
	20 µm	No treatment	80	0	10	10	20	20	10	50
		Silica coating	70	10	0	20	70	20	10	0
	50 µm	No treatment	80	20	0	0	80	20	0	0
		Silica coating	30	60	10	0	70	0	10	20
	100 µm	No treatment	100	0	0	0	100	0	0	0
		Silica coating	70	20	0	10	90	10	0	0
ND	Polished	No treatment	0	0	0	100	0	30	0	70
		Silica coating	20	0	0	80	0	0	0	100
	20 µm	No treatment	20	10	10	60	0	20	0	80
		Silica coating	30	20	0	50	10	30	0	60
	50 µm	No treatment	0	10	0	90	0	0	0	100
		Silica coating	0	0	10	90	0	0	0	100
	100 µm	No treatment	0	0	0	100	0	0	0	100
		Silica coating	0	0	0	100	0	0	0	100
GC	Polished	No treatment	0	40	0	60	30	20	0	50
		Silica coating	80	0	0	20	10	30	0	60
	20 µm	No treatment	20	10	0	70	40	20	0	40
		Silica coating	30	20	0	50	20	20	0	60
	50 µm	No treatment	80	0	0	20	60	0	0	40
		Silica coating	80	0	0	20	40	10	0	50
	100 µm	No treatment	20	10	0	70	0	20	0	80
		Silica coating	20	0	0	80	10	10	0	80
S	Polished	No treatment	0	0	10	90	30	0	0	70
		Silica coating	0	0	0	100	0	0	0	100
	20 µm	No treatment	0	10	0	90	0	10	0	90
		Silica coating	0	10	0	90	10	0	0	90
	50 µm	No treatment	0	10	0	90	0	0	0	100
		Silica coating	0	0	0	100	0	0	0	100
	100 µm	No treatment	0	0	0	100	0	10	0	90
		Silica coating	0	0	0	100	0	0	0	100

**Fig. 6:**
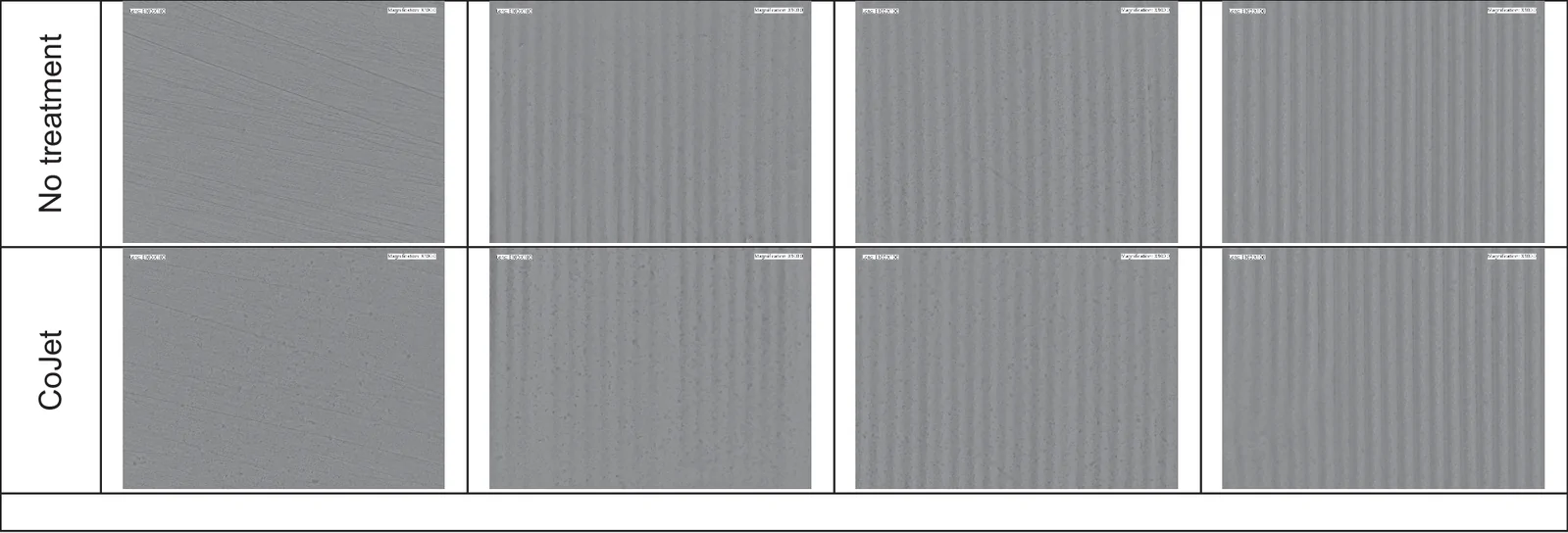
Representative digital microscopy images (100 ×) of the surface topography for the Delta material according to layer thickness (Polished, 20 μm, 50 μm, and 100 μm) and surface treatment (No treatment and silica coating). Untreated surfaces exhibited characteristic polishing marks, whereas silica coating introduced additional microdefects and surface irregularities. Printed specimens showed increasingly evident layer stratification with greater nominal layer thickness, which became more pronounced after silica coating

**Fig. 7:**
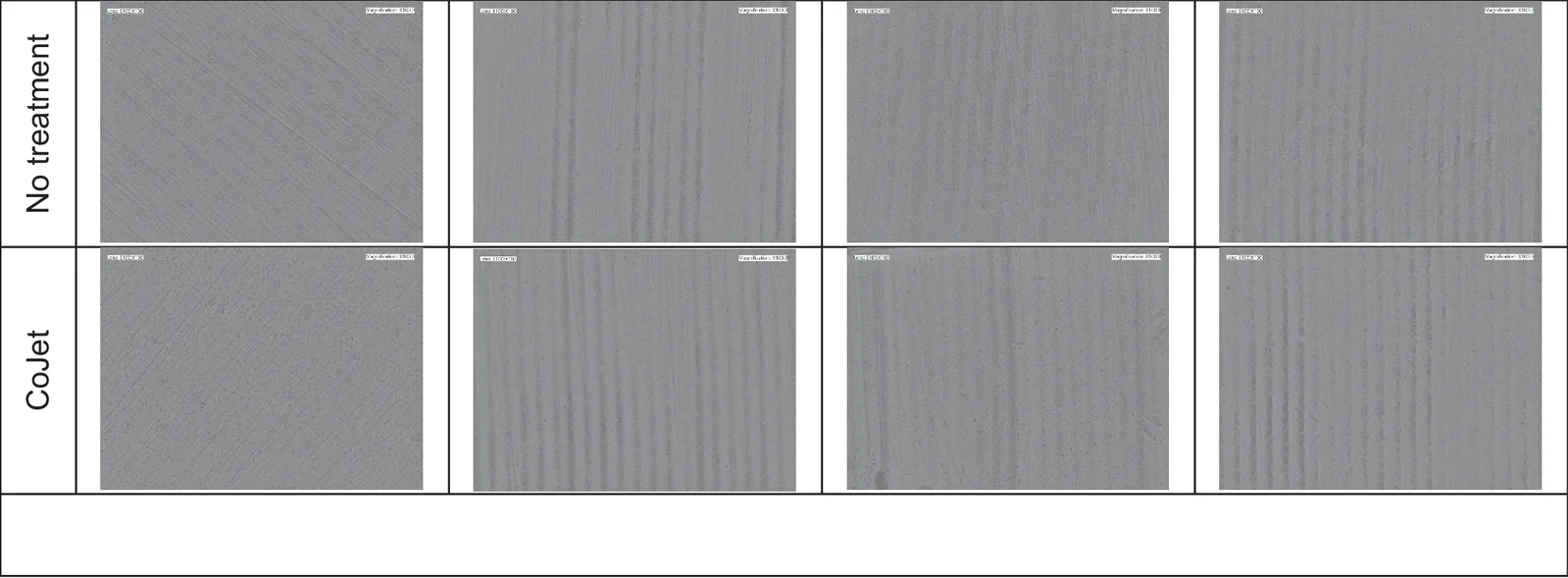
Representative digital microscopy images (100 ×) of the surface topography for the NextDent material according to layer thickness (Polished, 20 μm, 50 μm, and 100 μm) and surface treatment (No treatment and silica coating). In the untreated condition, printed layers were less distinct, particularly at lower layer thicknesses. After silica coating, layer stratification became clearly discernible, accompanied by increased surface irregularity

**Fig. 8:**
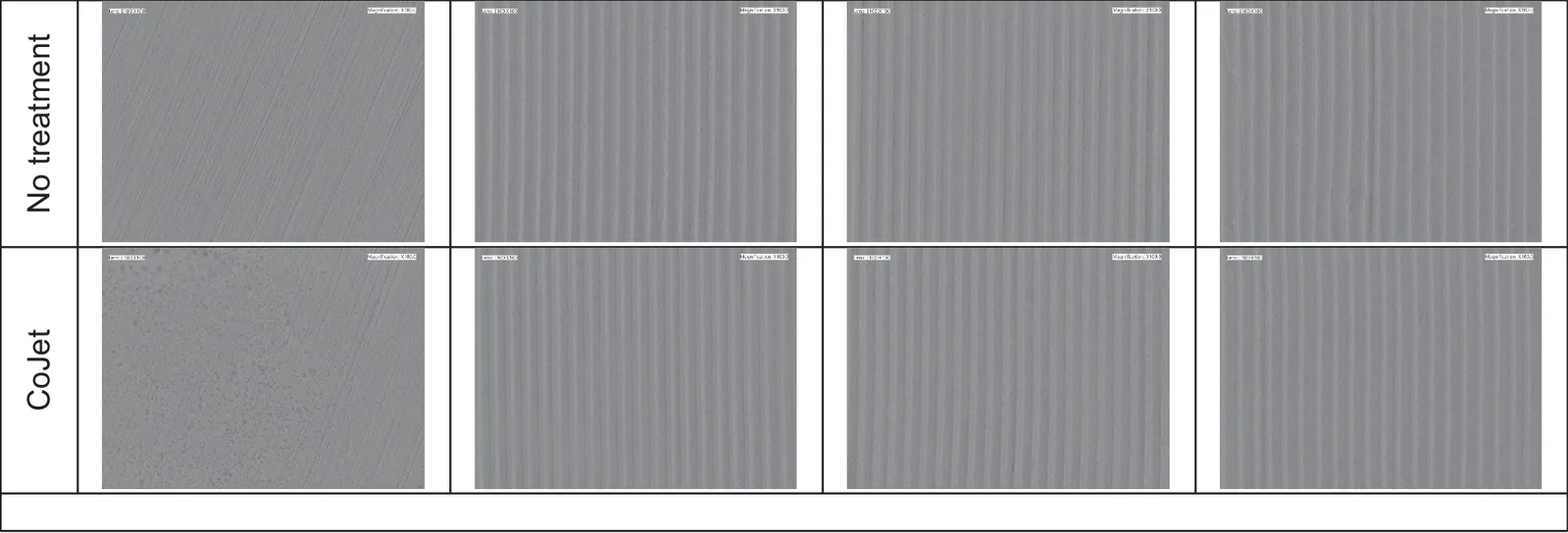
Representative digital microscopy images (100 ×) of the surface topography for the GC material according to layer thickness (Polished, 20 μm, 50 μm, and 100 μm) and surface treatment (No treatment and silica coating). Polished and untreated surfaces showed abrasion marks from surface finishing, while silica coating generated a more irregular topography with microdefects. Layer stratification became progressively more visible with increasing layer thickness, particularly after surface treatment

**Fig. 9:**
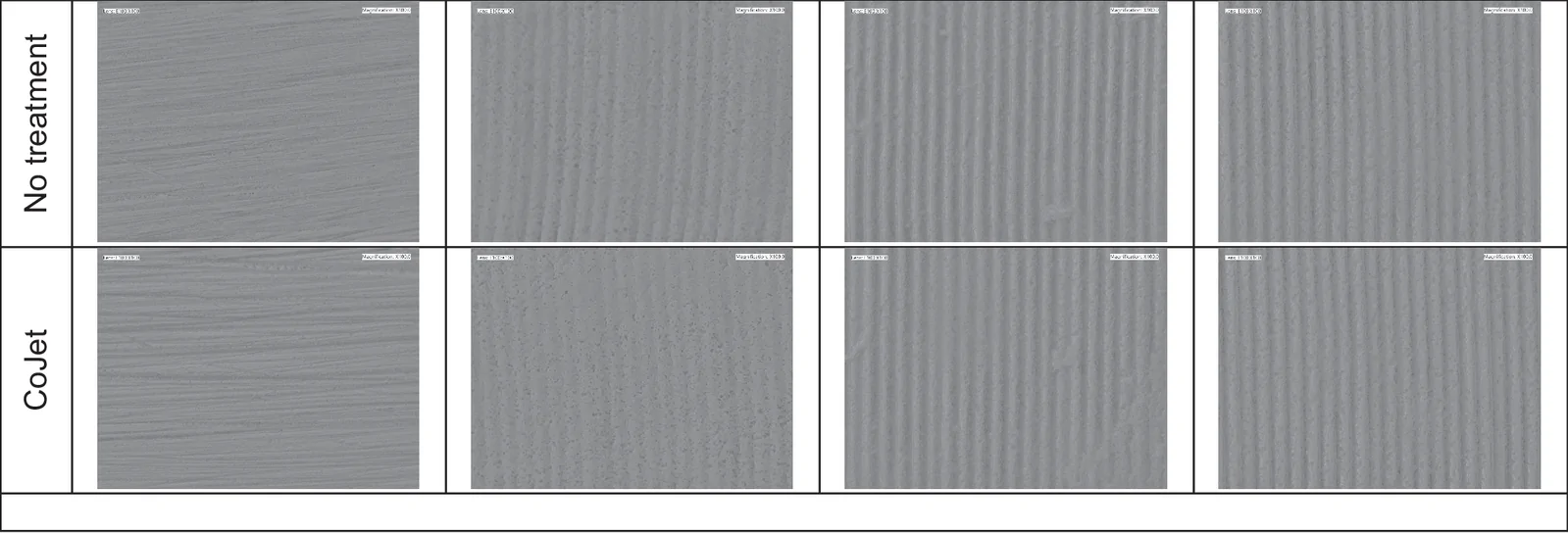
Representative digital microscopy images (100 ×) of the surface topography for the Saremco material according to layer thickness (Polished, 20 μm, 50 μm, and 100 μm) and surface treatment (No treatment and silica coating). Untreated surfaces exhibited smoother topographic features, whereas silica coating enhanced surface heterogeneity. Printed layers became more evident as layer thickness increased, with silica coating accentuating layer visibility while partially flattening surface peaks

Surface topography observations corroborated the quantitative roughness findings. Representative digital microscopy images of surface topography for all materials as a function of layer thickness and surface treatment are presented in Figs. 6, 7, 8 and 9. Untreated polished surfaces showed characteristic silicon carbide abrasion marks, whereas silica-coating surfaces exhibited additional microdefects and irregularities induced by air abrasion. In printed specimens, layer stratification became more pronounced with increasing nominal layer thickness, and this effect was further accentuated after silica coating, which enhanced layer visibility while partially flattening surface peaks. For ND material, the printed layers were less distinct in the untreated condition but became clearly discernible after silica coating.

## Discussion

The aim of this study was to evaluate how different 3D-printed resin composites, printed at various layer thicknesses and treated with or without silica-coating (CoJet), respond in terms of surface roughness, topography, and shear bond strength before and after thermocycling. The significant effects of material, layer thickness, and surface treatment, as well as their interactions, indicate that these factors influence surface characteristics and bonding performance. Accordingly, the null hypotheses were rejected for material type and layer thickness, while the effect of silica-coating was found to be condition dependent, confirming that 3D printing parameters, material composition, and surface treatment affect surface topography and adhesion, consistent with the premise that printed polymers are sensitive to structural and chemical modifications [4, 14, 16].

Layer thickness played a key role in determining the surface architecture of the printed resins, corroborating prior work showing that thinner layers produce smoother surfaces with fewer staircase effects, whereas thicker layers produce more pronounced stratification [6, 9, 10]. From a clinical perspective, printing resolution may also influence restoration accuracy, as thinner layers generally improve vertical resolution and surface continuity, which may contribute to better marginal adaptation, whereas thicker layers can increase the stair-step effect and surface irregularities that require additional finishing procedures [7–9]. In the present study, this layer-dependent effect was particularly evident in material D, where roughness increased progressively with layer thickness. Across materials, silica coating significantly increased roughness, consistent with its known ability to create microretentive features and expose a more reactive surface [15, 17, 18]. When roughness was related to adhesion outcomes, groups with initially smoother surfaces, particularly polished conditions, tended to show larger improvements after silica coating, supporting the expectation that increased roughness may enhance micromechanical interlocking; however, this effect was not consistently reflected in bond strength for printed layers that already exhibited substantial inherent surface irregularities [11, 12]. However, in printed groups already exhibiting substantial inherent irregularities, increases in roughness did not necessarily translate into higher SBS. This suggests that in additively manufactured resins, the staircase pattern and layer boundaries may already provide sufficient micromechanical retention, limiting the incremental benefit of further roughening. Thus, although roughness influences adhesive behavior, the relationship is nonlinear and strongly depends on each material's inherent printed microstructure.

Adhesion is not determined by surface features alone; the microscale structure and chemical composition of each material also play a decisive role. The evaluated 3D printed polymers differ substantially in their monomer systems and filler content, which influence polymer network formation during photopolymerization and post curing. In methacrylate based dental resins, monomer functionality, degree of conversion, and filler distribution govern the resulting cross-link density of the polymer matrix, which in turn affects hardness, water sorption, and resistance to hydrolytic degradation [1, 10, 24]. Materials forming highly cross-linked networks generally exhibit greater mechanical stability and lower water sorption, whereas less densely cross-linked matrices may be more susceptible to plasticization and interfacial degradation that can compromise bonding durability over time [1, 11].

Among the investigated materials, 3Delta Etemp contains UDMA and TMPTA, together with a relatively high fraction of silica and glass fillers, which may promote a rigid, highly cross-linked polymer structure. In contrast, NextDent C and B MFH consist mainly of methacrylic oligomers with lower filler content, potentially producing a more compliant polymer network that can accommodate interfacial stresses during thermal aging. GC Temp PRINT contains UDMA-based monomers combined with quartz fillers, while Saremco Crowntec incorporates Bis-EMA and PEG dimethacrylate derivatives, both of which may generate polymer matrices with different cross-link densities and water sorption behavior. These compositional differences may influence surface energy, chemical reactivity, and micromechanical interlocking with resin cements, ultimately affecting the durability of adhesion to additively manufactured restorations [11, 14, 16].

With respect to optimizing adhesion, silica coating was most beneficial for polished surfaces, where it effectively compensated for the lack of micromechanical retention and introduced silica to facilitate silane-mediated bonding [17, 18]. In printed groups, however, silica coating often did not yield significant improvements, as the intrinsic layered microstructure already provided sufficient irregularity. Regarding printing resolution, 20 µm emerged as the most favorable layer thickness across materials, often yielding higher bond strength and more consistent results, likely due to balanced surface uniformity and reduced step height [6, 9]. From a compositional standpoint, ND and GC printed at 20 µm showed the most favorable adhesion profiles, consistent with their more adaptable polymer matrices and controlled filler content, whereas D showed limited benefit from silica coating except in polished conditions, and S exhibited stable but not superior performance unless combined with silica coating on smooth surfaces.

From a clinical perspective, the magnitude of bond strength is as important as statistical significance. Laboratory studies often consider bond strengths of 10–13 MPa clinically acceptable for durable luting interfaces under controlled conditions [20]. In the present study, combinations like ND and GC printed at 20 µm achieved or approached this range, indicating potential for reliable bonding with proper surface conditioning. Lower values in polished or untreated conditions show that surface architecture and material composition significantly affect bonding. These findings suggest that print resolution and surface treatments should be chosen based on whether they produce bond strengths capable of supporting durable adhesive interfaces in prosthodontic restorations. However, these observations should be interpreted cautiously since the results were obtained in controlled in vitro conditions. Additional clinical studies and long-term durability tests are needed before making definitive clinical recommendations for optimal printing parameters or mandatory surface conditioning protocols.

Compared with previous studies on bonding to 3D-printed provisional resins, the present findings support the conclusion that adhesion is strongly material-dependent and not uniformly improved by a single surface treatment. Holmer et al. found that adhesive resin cement had higher bond strength than resin-modified glass ionomer cement on 3D-printed material, and that surface treatment effects depended on the luting system [26]. Lim and Shin showed that bonding varied with the repair material, with bisacryl composite exhibiting higher bond strength than methyl methacrylate-based PMMA, and that additional surface treatment didn't always help [11]. Soto Montero et al. reported that 3D-printed resins had higher microtensile bond strength than milled prostheses, with airborne particle abrasion being more effective for milled than for printed materials [27]. These results align with studies showing printed resin surfaces may already have enough micromechanical irregularity, so air abrasion doesn't always improve bond strength [11, 16, 22, 26, 27]. Discrepancies likely relate to differences in resin formulation, filler loading, degree of conversion, post curing, aging, cement chemistry, and bond test setup, affecting surface energy, polymer stability, and chemical vs. micromechanical bonding [14, 16, 22, 27].

Thermocycling influenced adhesive behavior in a material- and condition-dependent manner. Importantly, thermocycling did not uniformly reduce shear bond strength and, in several material–layer combinations, stabilized or slightly improved bonding performance, which may be attributed to continued post-polymerization, thermal relaxation, and increased cross-link density within the polymer matrix during aging [19, 24]. Printed materials with more flexible or less densely cross-linked matrices, such as ND and S, likely benefited from this maturation, explaining the improved or stable SBS values after aging. Conversely, polished untreated groups tended to show reductions in adhesion, consistent with hydrolytic degradation and the absence of micromechanical retention [23]. Thus, thermocycling did not uniformly weaken the interface; instead, its effect depended on the polymer network, the surface architecture, and the initial quality of interfacial interaction.

SBS evaluates only one type of interfacial stress and may not fully reproduce clinical loading conditions [20, 21]. Although thermocycling was used to simulate intraoral thermal aging, other factors such as mechanical fatigue and long-term water storage may also affect interfacial degradation and were not examined in the present study. Moreover, the simplified specimen geometry used in laboratory bond strength tests provides a controlled bonding area but does not replicate the complex morphology, curvature, and stress distribution present in clinical restorations such as crowns or inlays. Future investigations should incorporate additional surface treatments, such as universal adhesives, combined chemical–mechanical protocols, or optimized silane strategies, to determine whether chemical interactions can be enhanced in printed matrices with lower surface energy and reactive free monomers. Advanced interfacial analyses, including Raman spectroscopy, EDS, or nanomechanical mapping, may further clarify how polymer chemistry, filler distribution, and silica deposition influence bonding durability and support the development of evidence-based protocols for bonding resin cements to 3D-printed resin composites in prosthodontics.

## Conclusions

From this study, the following could be concluded:

1. Material type, layer thickness, and silica coating significantly affected the surface roughness and adhesion of resin cement to the 3D-printed resins tested.
2. Layer thickness affected surface topography and partially influences adhesion, with thinner layers producing more uniform surfaces.
3. Material composition plays a decisive role in bonding performance, as differences in polymer networks, filler types, and resin matrices affect interfacial interactions.
4. The most favorable adhesion outcomes in the present experimental conditions were observed for ND and GC printed at 20 µm.
5. Silica-coating was most beneficial for polished surfaces and showed limited or condition-dependent additional benefit for layered specimens, where inherent surface irregularities were already present.
6. Thermocycling did not uniformly degrade adhesion; in several material-layer combinations, it stabilized or slightly improved bond strength, whereas polished untreated specimens remained the most susceptible to hydrolytic degradation.

## Data Availability

No datasets were generated or analysed during the current study.
